# Supplementary material for: Roll-to-roll fabrication of touch-responsive cellulose photonic laminates
Source: Nat Commun. 2018 Nov 6;9:4632. doi: 10.1038/s41467-018-07048-6 (PMC6219516; doi:10.1038/s41467-018-07048-6)

Roll-to-roll fabrication of touch-sensitive cellulose photonic laminates

Liang, Bay *et al*.

Supplementary Information

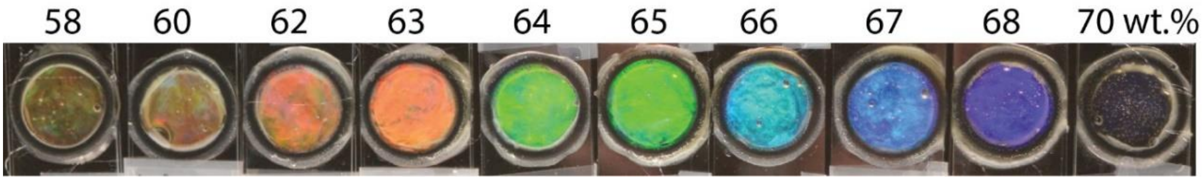

Supplementary Figure 1: Macroscopic images of HPC water mesophases of different concentrations (weight percent, wt.%) from left to right.

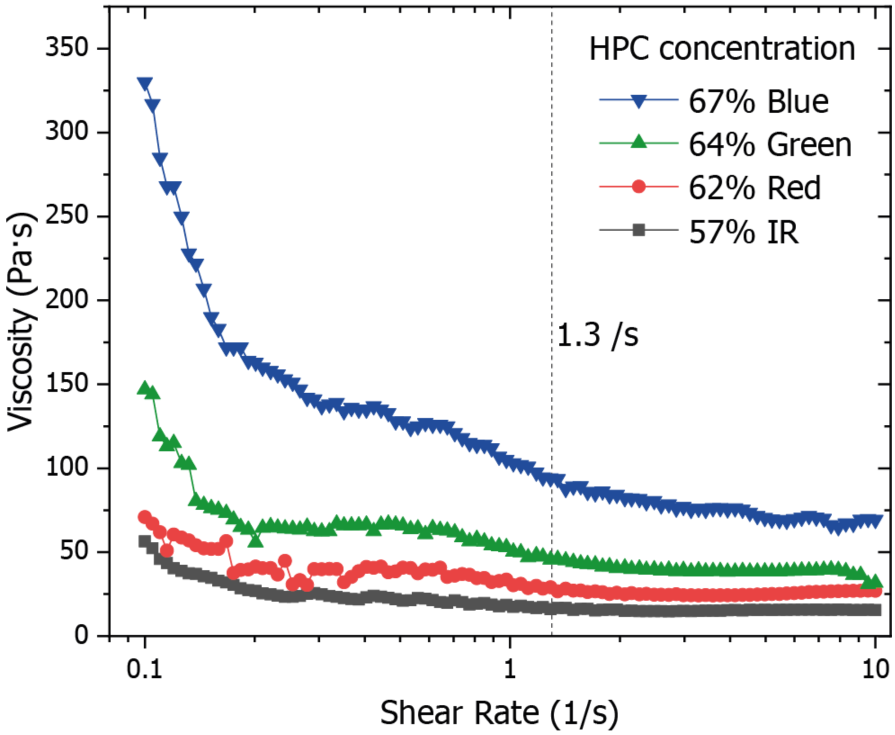

Supplementary Figure 2: Viscosity - shear rate profiles of HPC showing shear-thinning behaviour and increase of viscosity with concentration. Data obtained by flow sweep measurements using a commercial rheometer and 50 mm parallel plate geometry with a 1.2 mm gap and at 22°C to mimic the slot-die coating environment. A solvent trap was used to prevent the sample from drying during data acquisition. The shear rate was ramped from 0.1 s^{-1} to 10 s^{-1}, and three measurements made for each wt.% of HPC. The dashed line at 1.3 s^{-1} shear rate (similar to the slot-die process using 1.2 mm coating gap and 1.6 mm s^{-1} web speed) sits in the high-shear range where the viscosity of 57% HPC solution becomes independent of shear rate.

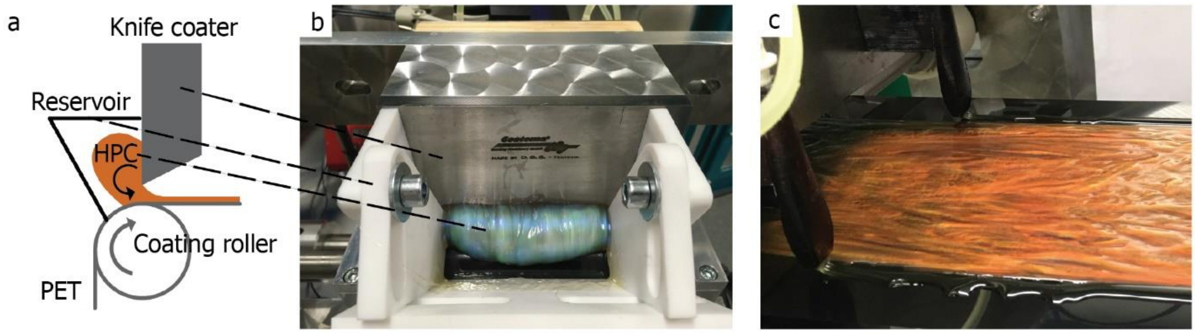

Supplementary Figure 3: HPC film surface instability associated with knife-over coating. **a – b,** Driven by the motion of the PET substrate, the HPC feed forms a tumbling vortex at subsequent knife shearing, causing flow disturbances which result in streak defects in the coating **(c)**.

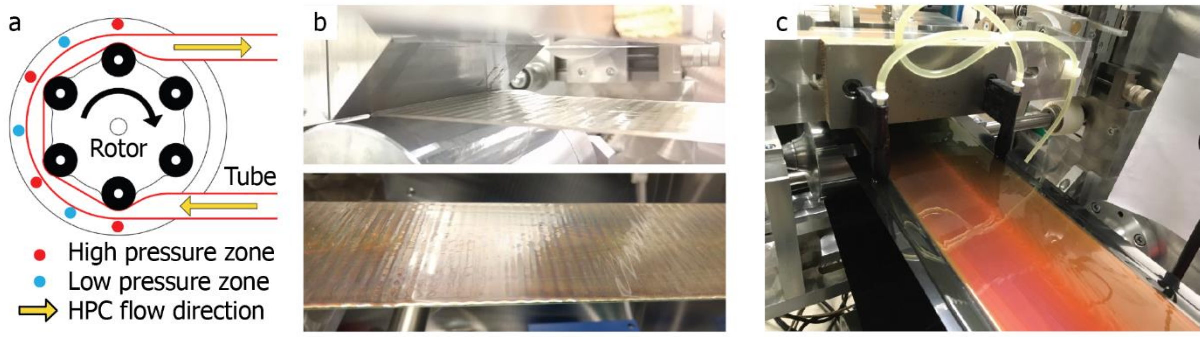

Supplementary Figure 4: a, Schematic of peristaltic pump: rotor periodically compresses tube to pump HPC inside. The intermittent pressure generated during fluid transport results in periodic fluctuations ("chatter" defects) in the HPC slot-die coating **(b).** Pulsation is reduced by adding a pressure dampener along the pump line (depicted in Fig. 1a), ensuring homogenous coating **(c).**

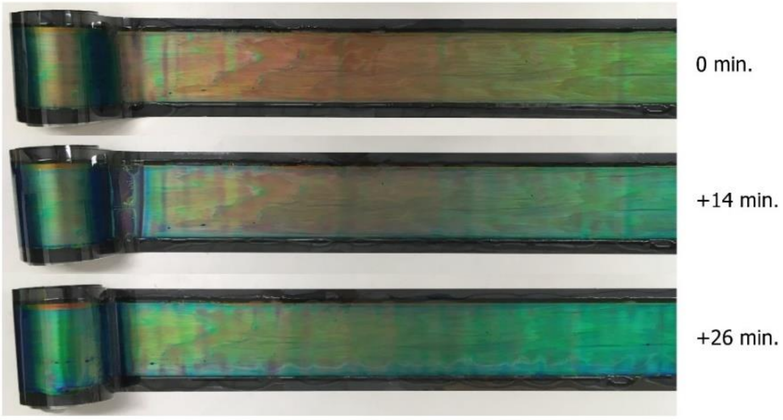

Supplementary Figure 5: Colour relaxation of a green HPC laminate as the stress in the mesophase from the fabrication process is relieved. Times are from completing the R2R fabrication. Characteristic colour is restored from orange to green in the final state at equilibrium.

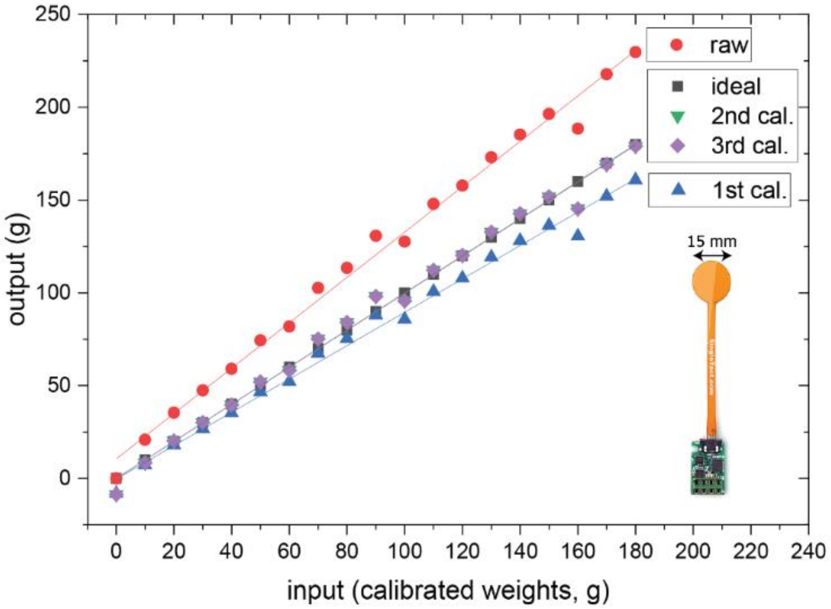

Supplementary Figure 6: Force sensor calibration. The force sensor (inset) is calibrated using a set of standard weights. Raw data show a linear trend with standard deviation 0.03. Calibration repeated until data matches the reference.

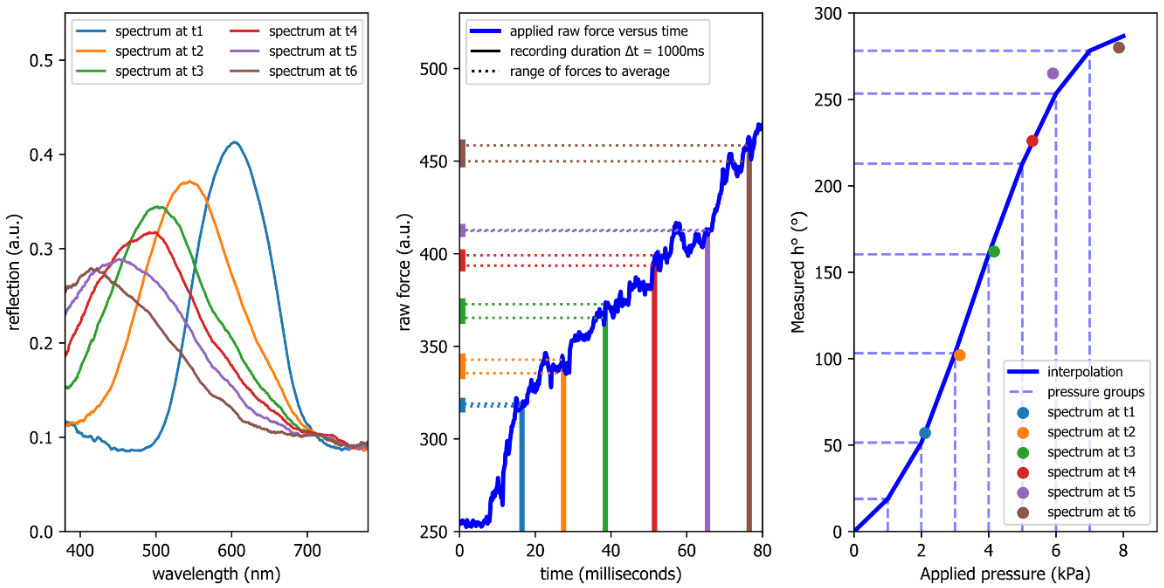

Supplementary Figure 7: Integrating sphere spectra with 1s integration (left plot, smoothed) as sample pressure is increased; end-time of each as indicated. Data from force sensor is simultaneously recorded every 400 ms, and force vs time is interpolated (middle plot). For each spectrum, the average force over each spectral acquisition (middle plot, coloured lines) is then converted to a calibrated pressure in kPa. The hue *h*° is calculated for each spectrum (right plot, coloured points), plotted against pressure (right plot, solid line) and interpolated. This interpolation (right plot, dotted lines) provides the pressure calibration between *h*° and pressure.

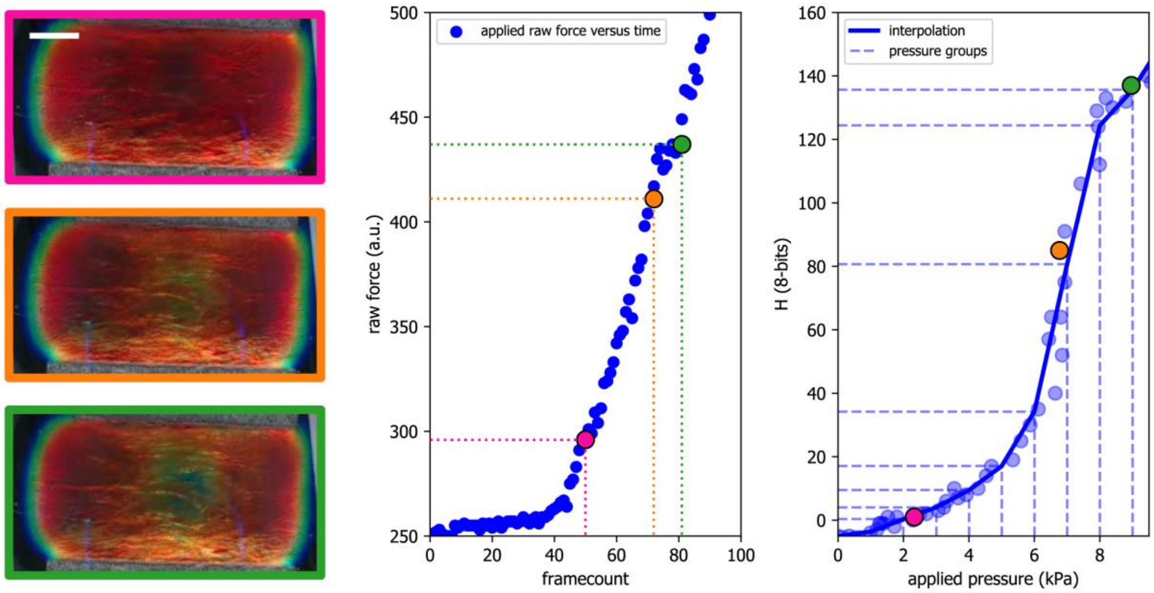

Supplementary Figure 8: On the camera set-up, video frames are recorded by the camera while the sample pressure is increased (exemplar frames on left). The force sensor reading every 400 ms triggers acquisition of both a video frame and force value (middle plot, showing 3 coloured points corresponding to the 3 photos). The force for each frame is converted to a calibrated pressure in kPa. The average hue *H* is calculated within the region of interest in each frame, plotted against pressure (right plot, points) and interpolated (right plot, solid blue line). This interpolation (right plot, dotted lines) provides the pressure calibration between *H* and pressure. (Scale bar: 10 mm)

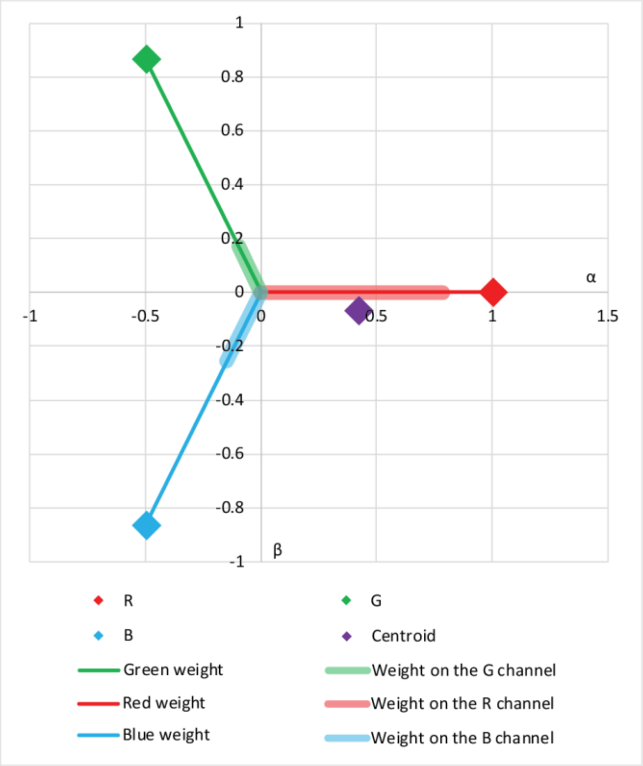

Supplementary Figure 9: Colours recorded by the camera consist of three 8-bits values for the three primary channels R (red), G (green) and B (blue). R is set as (255, 0, 0), G is set as (0, 255, 0) and B is set as (0, 0, 255), and a colour with coordinates r on the red channel, g on the green channel and b on the blue channel is represented by (r, g, b) in RGB coordinates, given by r × R + g × G + b × B.

On the RGB triangle plot, R, G and B vectors are set at a radius equal to 1 and at an angle of 120° from each other (the red, green and blue diamonds and lines), to make an equal re-partition. This is equivalent, in cartesian coordinates, to:

$$x_R = 1$$
$$y_R = 0$$
$$x_G = \cos(120°)$$
$$y_G = \sin(120°)$$
$$x_B = -\cos(120°)$$
$$y_B = -\sin(120°)$$

Every colour recorded in (r, g, b) coordinates is expressed in terms of its weight relative to each channel in cartesian coordinates:

$$\alpha = \frac{r \times x_R + g \times x_G + b \times x_B}{r + g + b} \quad (1)$$

$$\beta = \frac{r \times y_R + g \times y_G + b \times y_B}{r + g + b} \quad (2)$$

Each point representing a colour is therefore the centroid of the three primary colours R = (255, 0, 0), G = (0, 255, 0) and B = (0, 0, 255) normalized to 1 (for example, the purple diamond, with its weights r = 200, g = 50 and b = 70 represented as the black dots).

The closer a point is to one of the three primary points, the more weight this channel has compared to the other.

In the alternate *HSL* representation of RGB colours, *H* accounts for the hue and represents the shift of information from one channel to the other. In this RGB triangle, this shift is visible as a rotation around the triangle, moving from the red axis to the green axis to the blue axis.

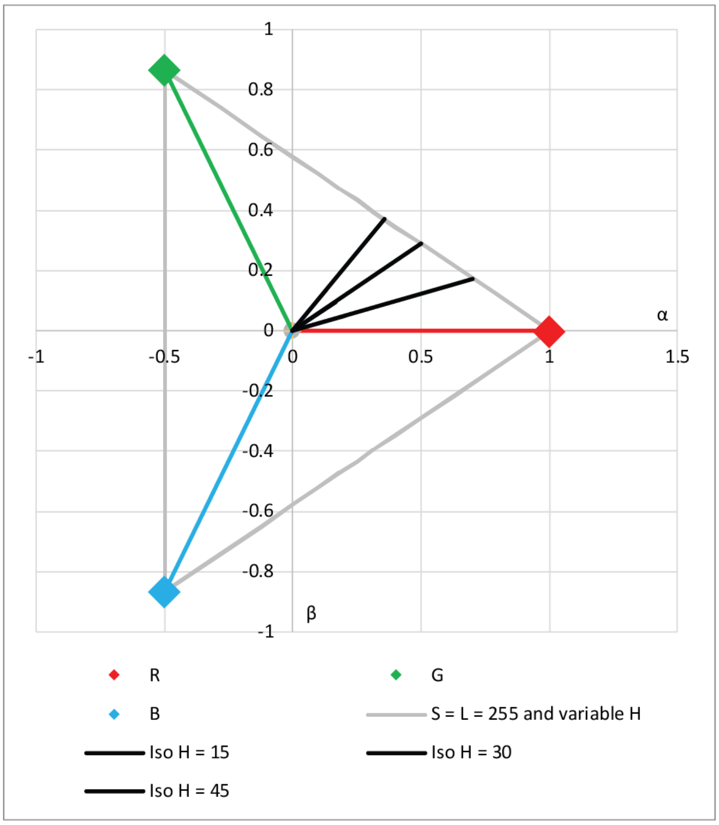

Supplementary Figure 10: Iso-*H* (all points that share the same H but have any possible S or L) are half-lines that start from the centre of the triangle. They are plotted in the form of dashed lines until their intersection with the triangle (no colour point can exist outside the triangle because all three primary channels are bounded at 255, or at 1 after normalisation). Therefore, the *H* boundaries corresponding to the pressure boundaries 0 - 10 kPa with 1 kPa increment can be represented on the perimeter of the RGB triangle and the position of the colour points allows one to read the corresponding *H*, or pressure.

Supplementary Movie 1

Full video of the foot pressure mapping shown in Fig. 4. An imprint process of total 5.5 s (right), performed by a young participant, was extracted into 166 frames for pressure mapping in false colour (middle). Trajectory of the hue (*H*) changes (right) based on the pressure change of the centre of the big toe (blue circle) is used for a dynamic analysis. As the participant retracts the foot from 2 s onwards, the decrease in pressure leads to the colour recovery towards the red.

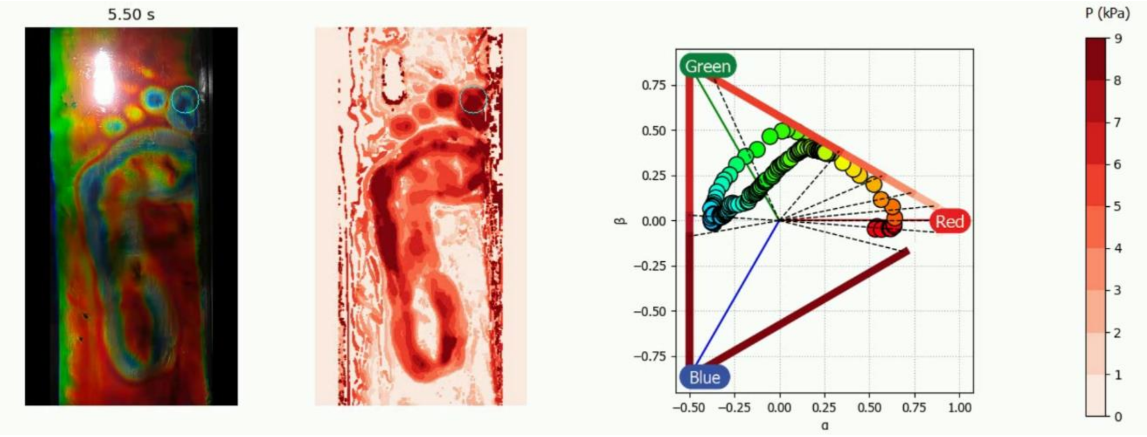

Supplement: Supplementary file 1 — Supplementary Information [file 41467_2018_7048_MOESM1_ESM.pdf]
