# Supplementary material for: Roll-to-roll fabrication of touch-responsive cellulose photonic laminates
Source: Nat Commun. 2018 Nov 6;9:4632. doi: 10.1038/s41467-018-07048-6 (PMC6219516; doi:10.1038/s41467-018-07048-6)
Supplement: Supplementary file 4 — Description of Additional Supplementary Files [file 41467_2018_7048_MOESM4_ESM.docx]

**Title:** Supplementary Movie 1
**Description:** Supplementary Movie 1 shows visually the main message of this work, which contains a full video of foot pressure mapping shown using our R2R fabricated HPC laminates. Dynamics of a footstep tracked over a total of 5.5 s performed by a young participant was extracted into 166 frames for pressure mapping in false colour. Trajectory of the hue (H) changes based on the pressure change of the centre of the big toe (blue circle) is used for a dynamic analysis. As the participant retracts their foot from 2 s onwards, the decrease in pressure leads to colour recovery towards the red.
